# Preparation and Phase Transition Study of a Multi-Environmentally Sensitive Hydrogel

**DOI:** 10.3390/gels12090793

**Published:** 2026-09-01

**Authors:** Bao Wang, Fengya Liu, Liqiu Zhai, Yuanyuan Zhang, Haijuan Tian, Yan Pan, Fengqi Liu

**Affiliations:** 1 College of Grain Engineering and Nutritional Science, Jilin Business and Technology College, Changchun 130507, China; 2 College of Chemistry, Key Laboratory of High Performance Plastics, Ministry of Education, Jilin University, Changchun 130012, China; 3 China Mobile Communications Group Co., Ltd., Changchun 130507, China

**Keywords:** hydrogel, stimuli-responsive, phase transition, pH-responsive

## Abstract

Hydrogels have attracted considerable attention due to their ability to undergo rapid and reversible phase transitions in response to external stimuli. In this work, a novel multi-responsive hydrogel (PT-Gel) was synthesized via the crosslinking of a linear poly(PEGDGE-co-PPGDGE) prepolymer (designated polyPEMPP, where PEMPP stands for Poly(Ethylene glycol) diglycidyl ether-co-Poly(Propylene glycol) diglycidyl ether) with trimethylolpropane tris(2-methyl-1-aziridinepropionate) (T-403), a trifunctional amine crosslinker. The phase transition behavior was evaluated by UV–Vis transmittance measurements at 600 nm as a function of temperature. The phase transition temperature (T_T_) of PT-Gels can be precisely tuned from 11.8 ± 0.5 °C to 76.3 ± 1.2 °C by adjusting the hydrophilic poly(ethylene glycol) diglycidyl ether (PEGDGE) content (0–35%), pH (5.0–10.5), NaCl concentration (0–0.9 wt%), and urea concentration (0–0.7 wt%). Specifically, T_T_ exhibits a nearly linear positive correlation with PEGDGE content and a linear negative correlation with pH, decreasing by 4.8 ± 0.6 °C per pH unit. The addition of 0.9 wt% NaCl lowers T_T_ by 11.7 ± 0.8 °C through charge screening, while 0.7 wt% urea elevates T_T_ by 36.5 ± 1.5 °C through hydrogen bond disruption. For the optimal formulation (15% PEGDGE, pH 7.7), T_T_ and transition width (ΔT) are 34.2 ± 0.6 °C and 4.2 ± 0.3 °C, respectively. PT-Gels demonstrate excellent reversible phase transition behavior, maintaining stable transmittance cycles between 98.7 ± 0.5% and 1.2 ± 0.3% over five consecutive heating–cooling cycles without significant degradation. The main driving forces for the phase transition are identified as hydrophobic interactions, electrostatic repulsion, and hydrogen bonding. This proof-of-concept study provides a rational design strategy for multi-responsive hydrogels with tunable phase transition for applications in smart drug delivery, sensors, and separation systems, while the current limitations include the need for further structural characterization and mechanical property evaluation for specific biomedical applications.

## 1. Introduction

Stimuli-responsive hydrogels have been extensively studied over the past few decades due to their ability to undergo significant and rapid changes in volume, shape, or optical properties in response to external stimuli such as temperature, pH, ionic strength, and electric fields [1,2,3,4]. These unique characteristics have spurred worldwide research into their applications in drug delivery [5,6], chemical separation [7], tissue engineering [8,9,10,11,12], and sensors [13,14,15]. Among the various stimuli, temperature is one of the most commonly used triggers due to the ease of controlling and designing temperature changes [16,17]. Temperature-responsive hydrogels can be broadly classified into two categories: negative thermoresponsive hydrogels exhibiting a lower critical solution temperature (LCST) and positive thermoresponsive hydrogels exhibiting an upper critical solution temperature (UCST) [18]. Poly(N-isopropylacrylamide) (PNIPAM) hydrogels are a typical example of LCST-type gels, for which the phase transition is driven by hydrophobic interactions [19,20,21,22]. Positive thermoresponsive systems, which exhibit an upper critical solution temperature (UCST), are often realized through interpenetrating polymer networks (IPNs) or copolymer systems containing complementary hydrogen-bonding pairs, where the phase transition behavior is driven by temperature-dependent hydrogen bonding association and dissociation [18,23,24,25].

In addition to temperature, pH-responsive hydrogels have also been widely investigated due to their potential in controlled drug delivery and biomedicine [26,27,28]. pH-responsive hydrogels typically contain ionizable groups such as amines or carboxylic acids that undergo protonation or deprotonation in response to changes in pH, leading to changes in the network structure and swelling behavior [29,30]. The combination of temperature and pH responsiveness in a single hydrogel system can provide additional flexibility for designing smart materials [31,32,33]. Given that our PT-Gel system contains tertiary amine groups that can be protonated or deprotonated depending on pH, understanding pH-responsive behavior is essential for predicting and tuning the phase transition properties of the hydrogel under physiologically and environmentally relevant conditions.

Recent advances have focused on designing systems with dynamic and reversible properties. For instance, Schiff base bonds have been extensively utilized to create self-healing and pH-responsive hydrogels, particularly for wound healing and drug delivery applications [34,35]. Furthermore, the effects of salts and urea on the phase transition behavior of hydrogels have been explored to understand the underlying mechanisms [36,37]. Salts can induce charge screening effects, which influence the electrostatic interactions within the gel network [38], while urea can disrupt hydrogen bonds, thereby affecting the hydrophobic interactions that drive the phase transition [39,40,41].

In the field of advanced intelligent systems, stimuli-responsive materials have also demonstrated remarkable potential in soft robotics and biosensing platforms [42,43]. For instance, soft magnetic robots with switchable morphologies have been developed for multimodal locomotion [42], while hydrogen bonding-driven hydrogels have been employed for real-time human motion monitoring [43]. These emerging applications further highlight the importance of understanding the fundamental driving forces—such as hydrogen bonding, hydrophobic interactions, and electrostatic effects—that govern the responsive behavior of smart hydrogels.

Despite these advances, several critical limitations remain in the design and application of multi-responsive hydrogels. First, most existing systems exhibit a narrow tunable range of T_T_, restricting their adaptability to different application scenarios. Second, although many hydrogels respond to individual stimuli, systematic and quantitative investigations of the combined effects of multiple stimuli—particularly the simultaneous influence of pH, salts, and urea—are scarce. Third, the underlying structure–property relationships governing the phase transition behavior are often insufficiently understood, hindering the rational design of hydrogels with predictable performance. Fourth, many multi-responsive hydrogels suffer from poor reversibility and cycling stability, which limit their practical utility in long-term applications. To address these challenges, we designed a novel PT-Gel system with the following hypothesis: the phase transition behavior of crosslinked polyPEMPPs(poly(poly(ethylene glycol) diglycidyl ether—monoethanolamine—poly(propylene glycol) diglycidyl ether))-based hydrogels can be systematically tuned by adjusting the hydrophilic/hydrophobic balance, pH, ionic strength, and urea concentration, driven by the synergistic interplay of hydrophobic interactions, electrostatic repulsion, and hydrogen bonding. The novelty of this work lies in the following: (1) the synthesis of a novel hydrogel via a simple one-pot crosslinking strategy; (2) the establishment of quantitative correlations between composition and T_T_ (e.g., near-linear positive correlation with PEGDGE content); (3) the comprehensive and quantitative investigation of four different stimuli (PEGDGE, pH, NaCl, urea) within a single system; (4) the systematic decoupling and identification of the three main driving forces governing the phase transition. By bridging these research gaps, this study provides a rational design strategy for multi-responsive hydrogels with precisely tunable phase transitions and excellent reversibility, offering significant potential for applications in smart drug delivery, sensors, and separation systems. On the basis of our previous work, in which we synthesized a series of linear polyPEMPP prepolymers and investigated their thermoresponsive behavior [44,45], we herein designed a novel hydrogel system (PT-Gels) by crosslinking polyPEMPPs with T-403, a trifunctional amine crosslinker. In this study, we systematically investigated the effects of hydrophilic segment content (PEGDGE), pH, NaCl, and urea on the phase transition behavior of PT-Gels, with particular emphasis on the phase transition temperature (T_T_), transition width (ΔT), and reversibility. The main driving forces for the phase transition were identified, and the quantitative correlations between composition and T_T_ were established.

## 2. Results and Discussion

### 2.1. Network Structure of PT-Gels

**Scheme 1:**
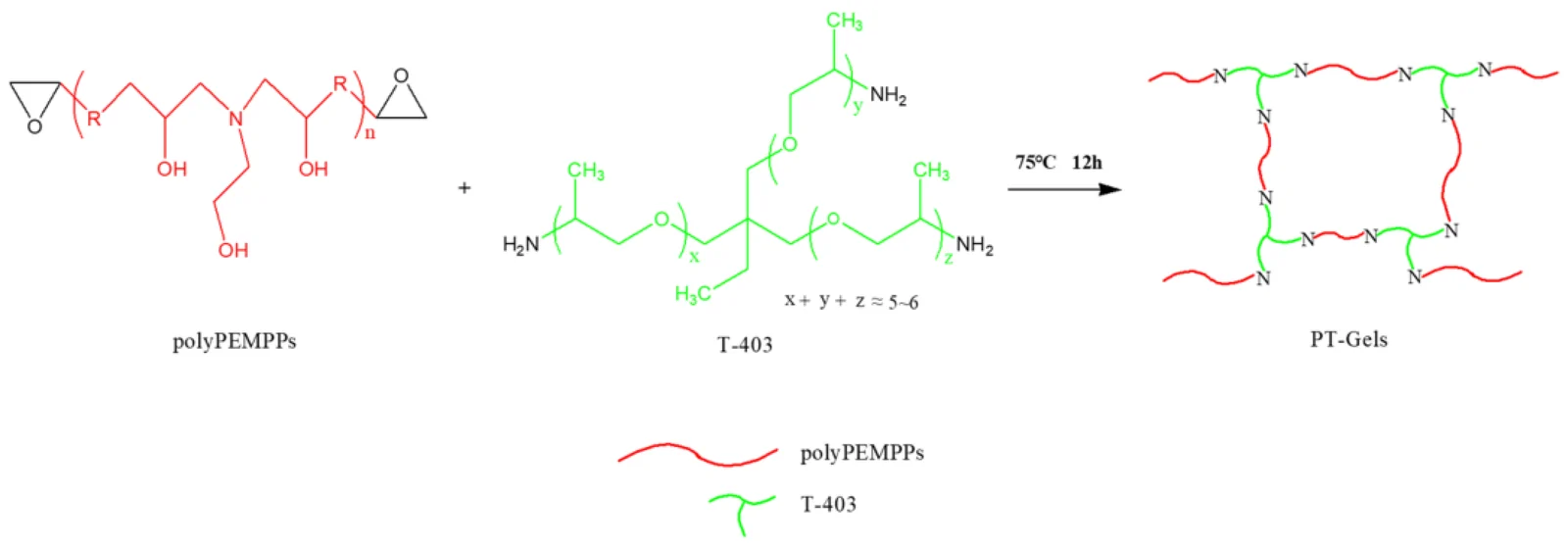
Synthesis route of PT-Gels via crosslinking of polyPEMPP prepolymer with T-403.

**Scheme 2:**
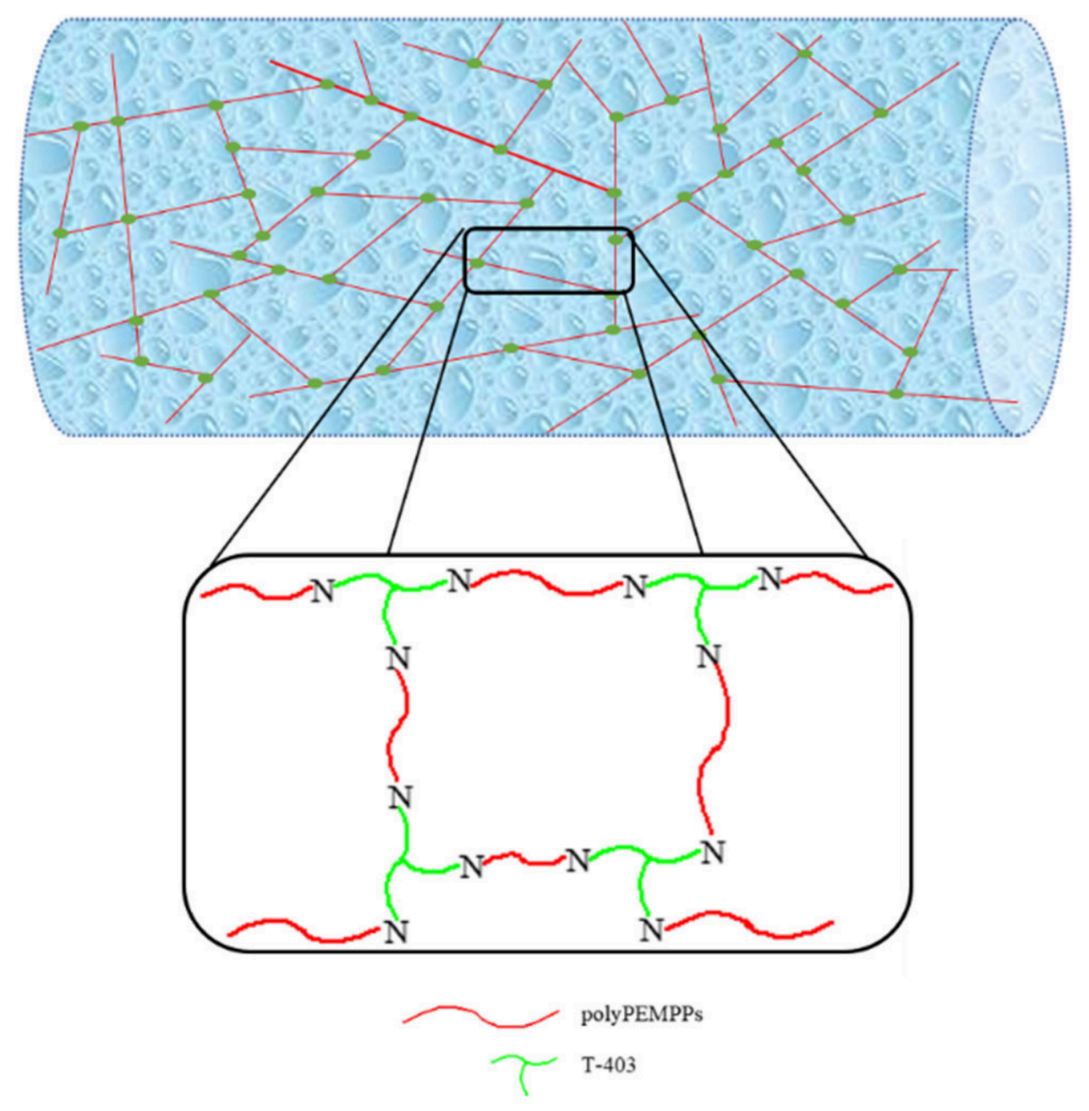
Schematic illustration of the three-dimensional crosslinked network structure of PT-Gels.

Scheme 1 illustrates the structural model of PT-Gels. The crosslinker T-403 is a trifunctional primary amine with an average molecular weight of 440, and its three primary amine groups can undergo nucleophilic addition/ring-opening reactions with the terminal epoxy groups of polyPEMPPs. Thus, each T-403 molecule connects three polyPEMPP chains, forming a typical chemically crosslinked hydrogel. The long polyPEMPP chains between crosslinks contain hydrophilic PEGDGE segments and hydrophobic poly(propylene glycol) diglycidyl ether (PPGDGE) segments. At elevated temperatures, the PPGDGE segments undergo hydrophobic contraction, leading to local variations in density. This causes differences in refractive indices, resulting in a macroscopic phase transition from transparent to opaque, which is reversible.The three-dimensional spatial distribution of the hydrophilic and hydrophobic segments, as well as the crosslinking junctions, is schematically illustrated in Scheme 2.

### 2.2. Effect of PEGDGE Content on Phase Transition

**Figure 1:**
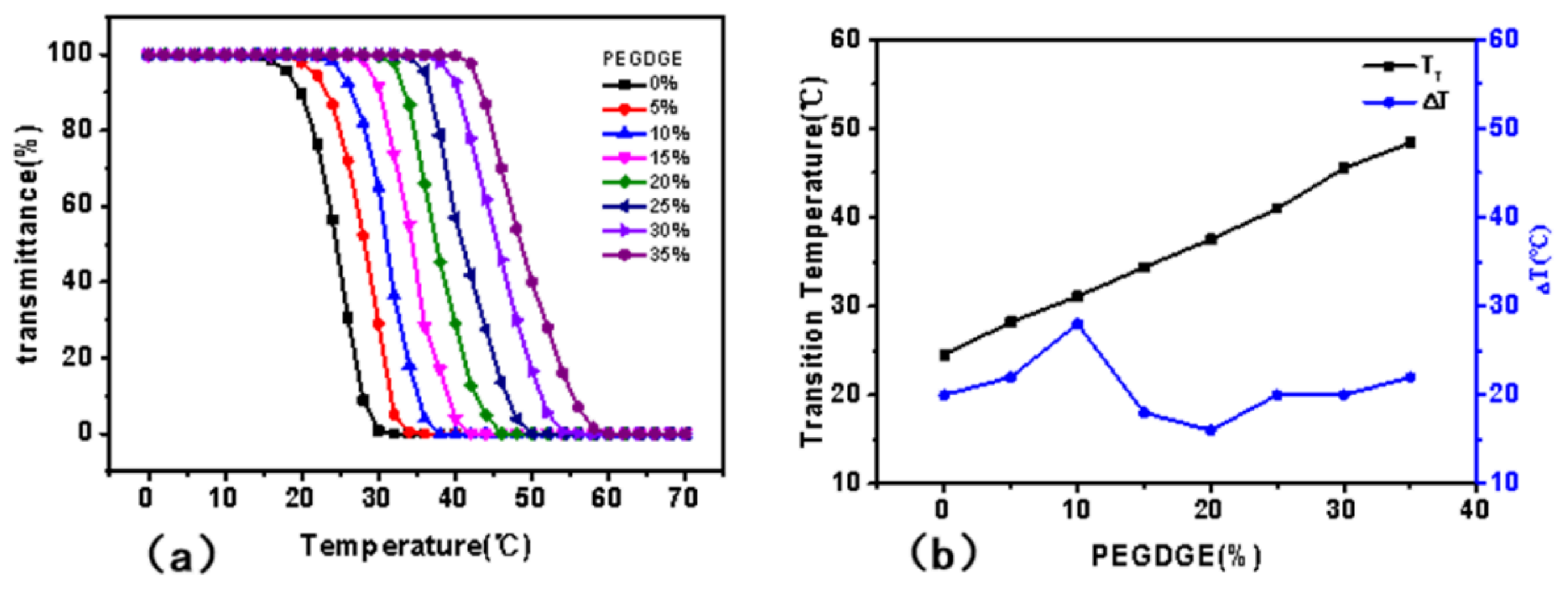
Influence of PEGDGE content on the phase transition temperature: (**a**) dependence of transmittance on PEGDGE content; (**b**) dependence of temperature (transmittance = 75%) on PEGDGE content.

Figure 1 shows the influence of PEGDGE content on the phase transition temperature. When PEGDGE content increases from 0% to 35%, the phase transition temperature (T_T_) increases. This is because more hydrophilic segments enhance the overall hydrophilicity of the gel, making hydrophobic association more difficult, thus requiring a higher temperature to induce phase transition. This is consistent with the recent findings on amphiphilic copolymers, where increasing the hydrophilic component enhances the overall hydrophilicity of the gel, thereby requiring a higher temperature to induce hydrophobic collapse [46,47]. Figure 1b indicates a nearly linear relationship between T_T_ and PEGDGE content, while the temperature range of the transition (ΔT) shows no clear linear trend.

### 2.3. Effect of pH on Phase Transition

#### 2.3.1. Effect of HCl

**Figure 2:**
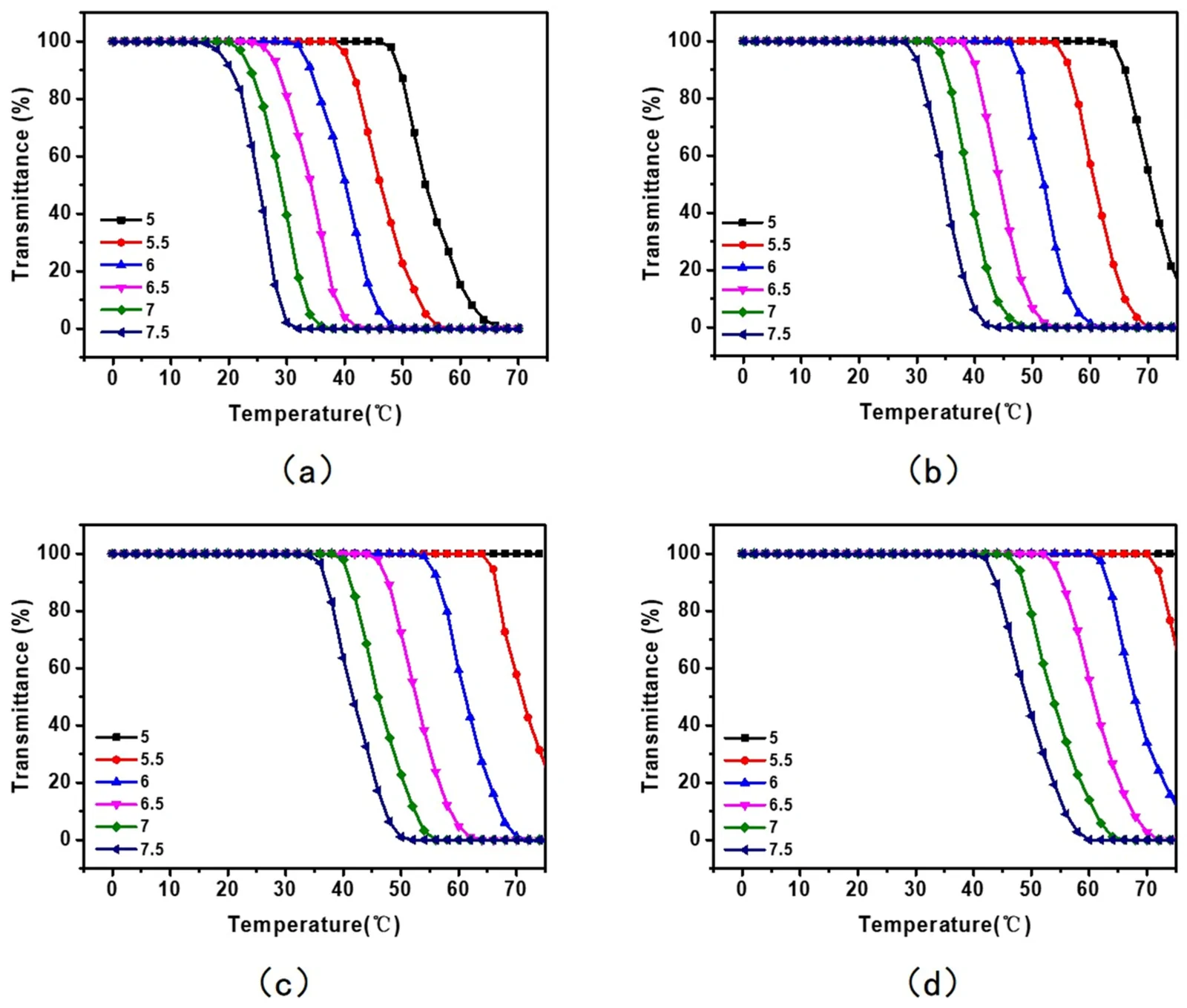
Phase transition behavior of PT-Gels with different PEGDGE contents at different pH (5–7.5): (**a**) PEGDGE = 0%; (**b**) PEGDGE = 15%; (**c**) PEGDGE = 25%; (**d**) PEGDGE = 35%.

**Figure 3:**
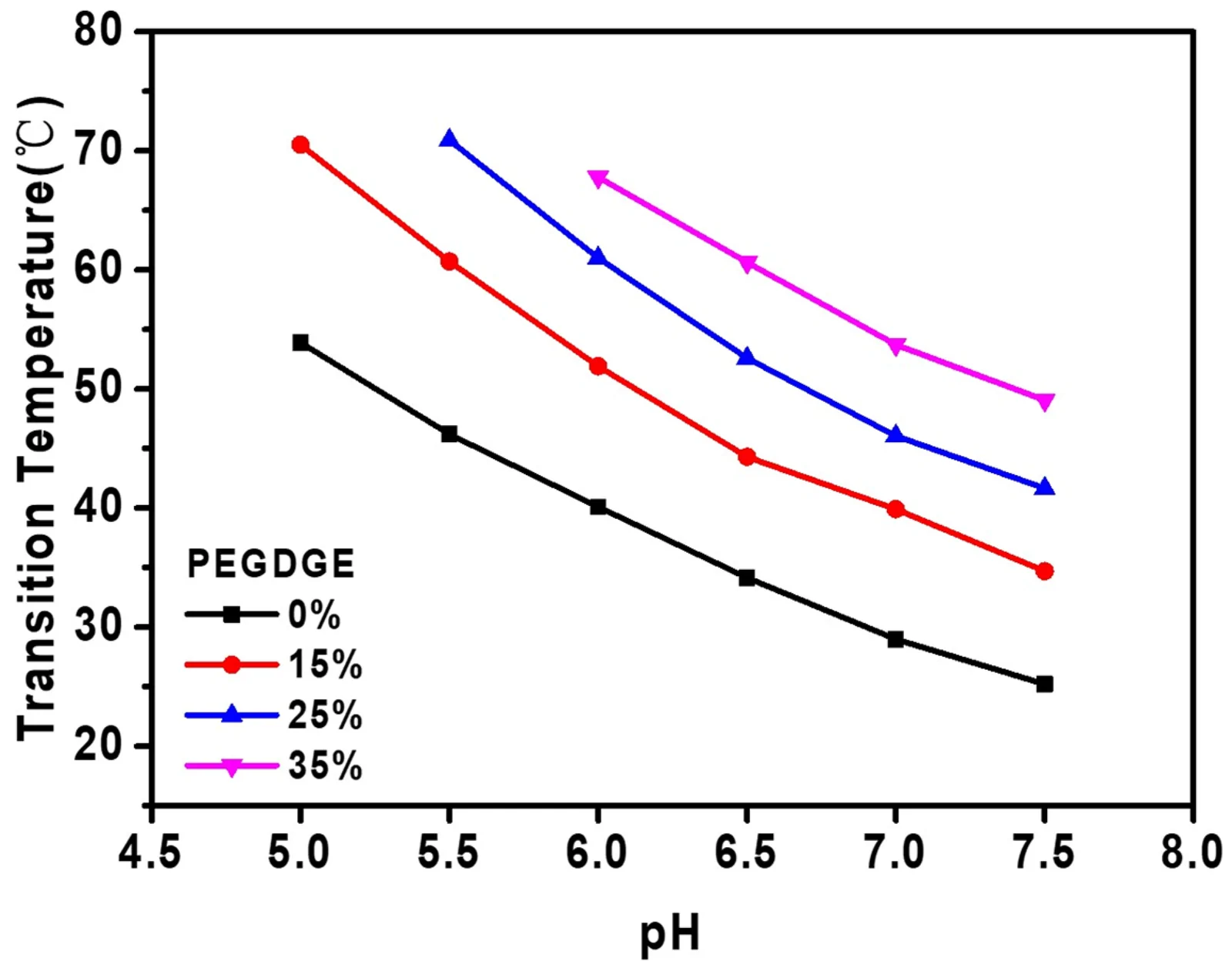
T_T_ and pH dependence of PT-Gels with different PEGDGE content.

PT-Gels contain tertiary amine groups that can be protonated or deprotonated depending on pH. Figure 2 displays the phase transition behavior of PT-Gels with different PEGDGE contents at pH values from 5 to 7.5. As pH decreases, T_T_ increases. Two reasons are proposed: (1) the protonation of tertiary amines introduces positive charges, generating electrostatic repulsion that hinders hydrophobic association; (2) charged chains become more hydrated, increasing hydrophilicity. Both factors raise T_T_. Figure 3 shows a linear negative correlation between T_T_ and pH. For higher PEGDGE contents (e.g., 35%), T_T_ becomes too high to be measured at low pH (e.g., pH 5.5). This aligns with the design principles of pH-responsive polymers, where charge density directly impacts the swelling and transition behavior [48,49].

**Figure 4:**
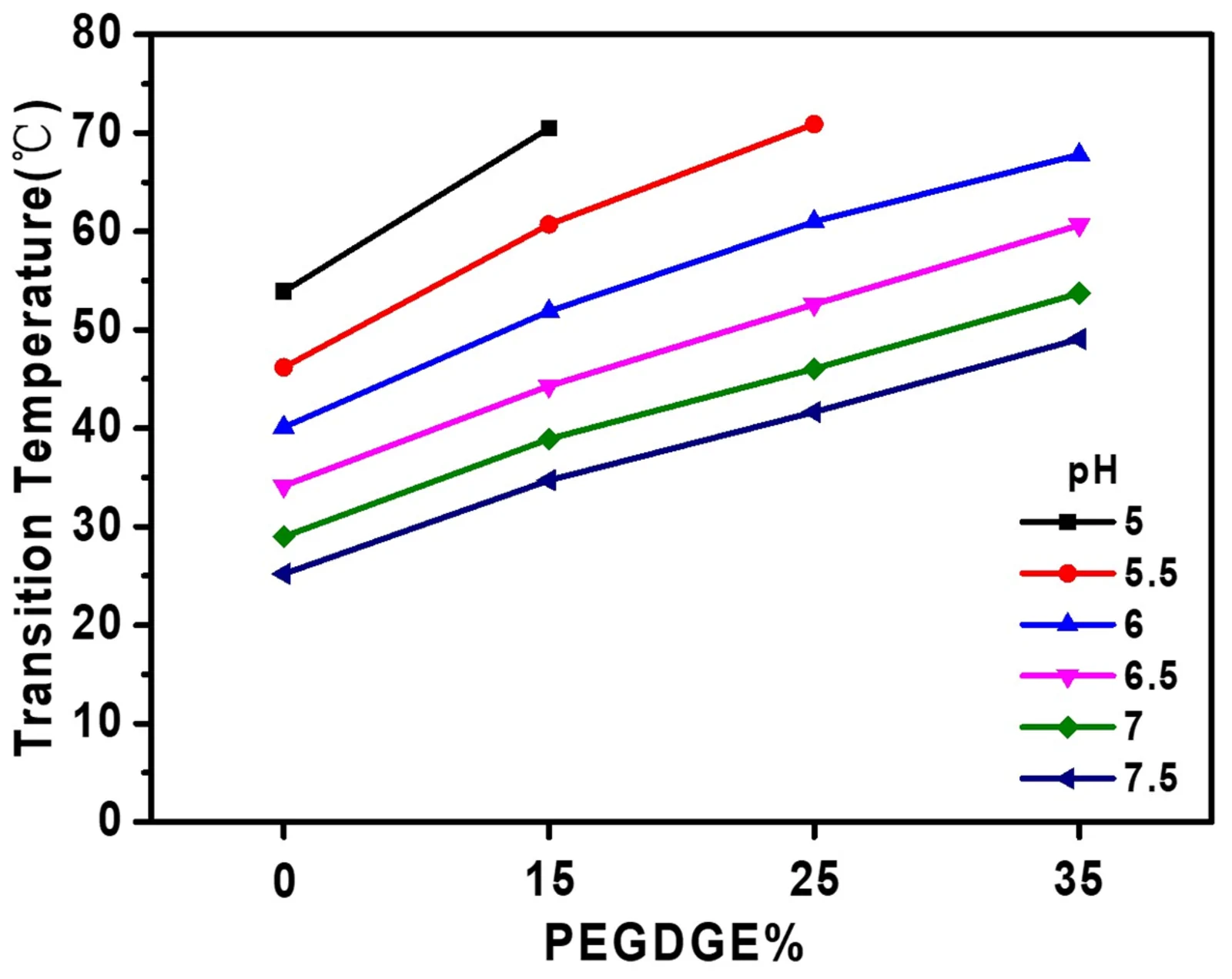
T_T_ and PEGDGE content dependence of PT-Gels with different pH.

Figure 4 plots T_T_ versus PEGDGE content at different pH values. For pH 5 and 5.5, T_T_ cannot be measured when PEGDGE exceeds 15% and 25%, respectively. T_T_ could be measured in experiments of the other groups. The overall relationship is that as the PEGDGE content increases, T_T_ increases. The missing data points at pH 5 and 5.5 are attributable to the fact that under these acidic conditions, the tertiary amine groups within the gel network are fully protonated, rendering the hydrogel highly hydrophilic. Consequently, as the PEGDGE content increases (exceeding 15% at pH 5 and 25% at pH 5.5), the phase transition temperature is elevated beyond the maximum measurable temperature (75 °C) limited by the water-bath heating system used in our transmittance measurements. Therefore, these data points could not be recorded within the instrumental range.

#### 2.3.2. Effect of NaOH

**Figure 5:**
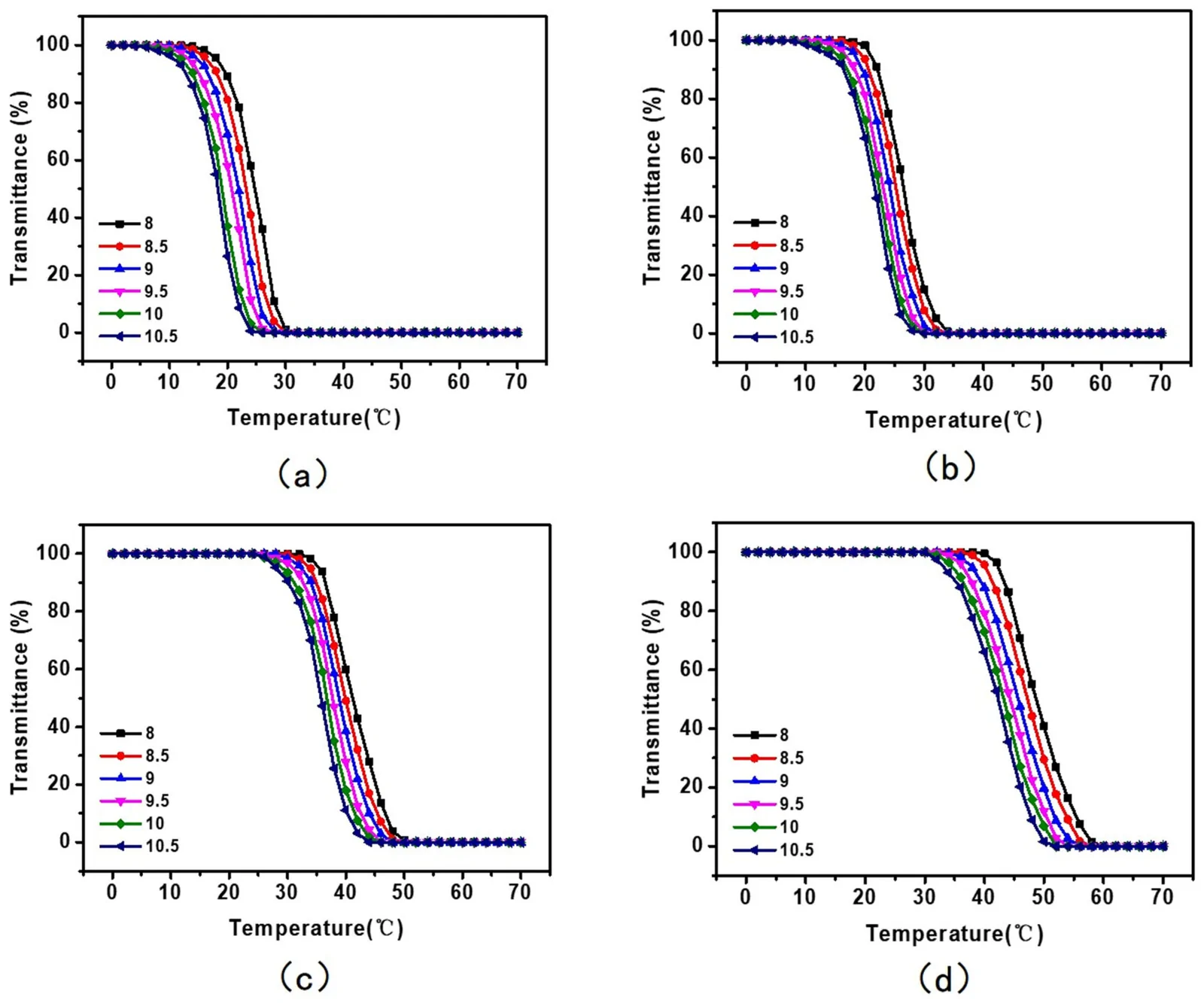
Phase transition behavior of PT-Gels with different PEGDGE contents at different pH (8–10.5): (**a**) pH dependence of the optical transmittance for PT-Gels with PEGDGE = 0%; (**b**) pH dependence of the optical transmittance for PT-Gels with PEGDGE = 15%; (**c**) pH dependence of the optical transmittance for PT-Gels with PEGDGE = 25%; (**d**) pH dependence of the optical transmittance for PT-Gels with PEGDGE = 35%.

Figure 5 shows the phase transition behavior at pH 8–10.5. As pH increases, T_T_ decreases. Adding OH^−^ ions promotes the deprotonation of tertiary amines, reducing electrostatic repulsion and facilitating hydrophobic association. Compared to HCl, NaOH has a weaker effect on the phase transition because adding a base does not form ammonium salts; it only makes the system more neutral.

**Figure 6:**
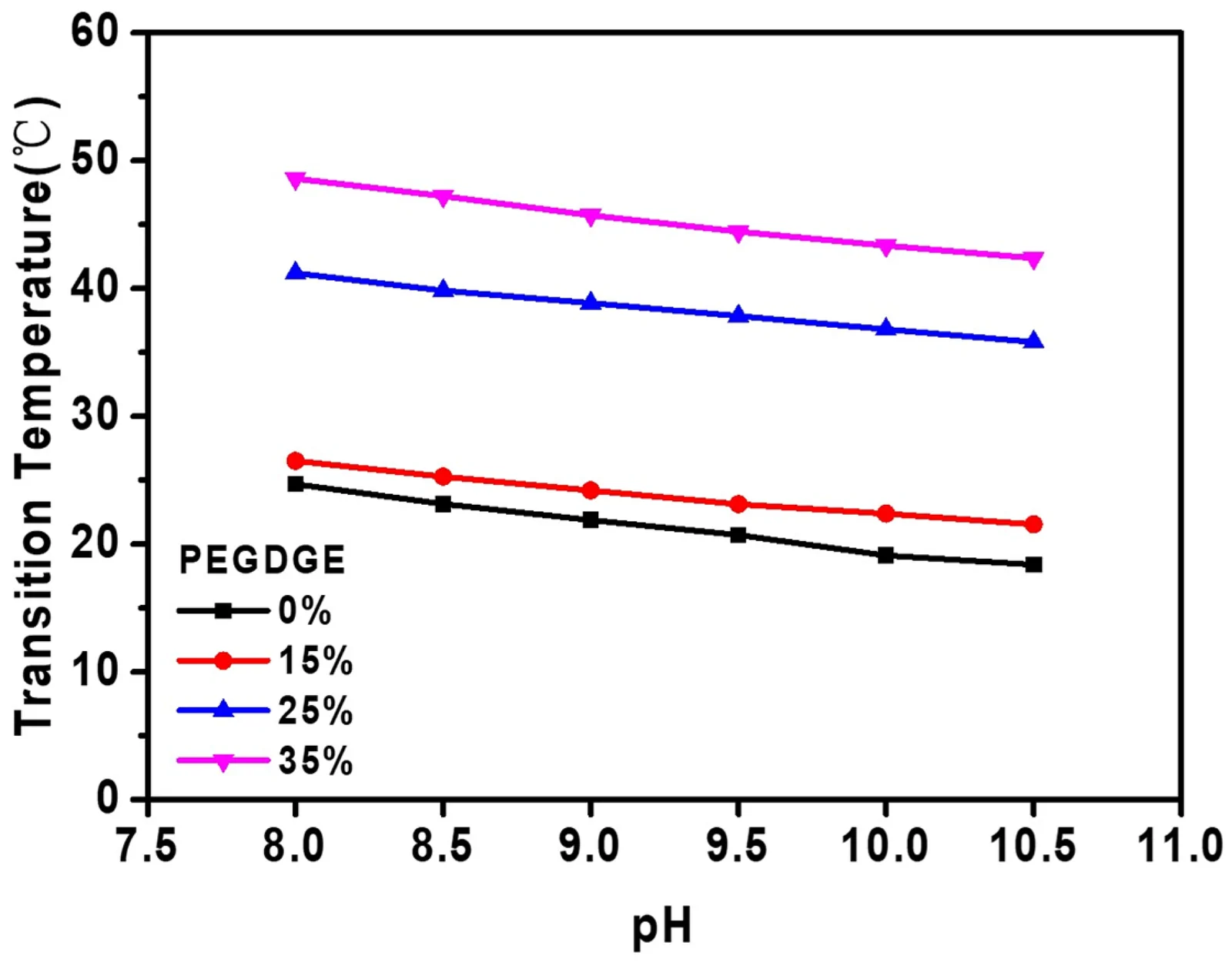
T_T_ and pH dependence of PT-Gels with different PEGDGE content.

**Figure 7:**
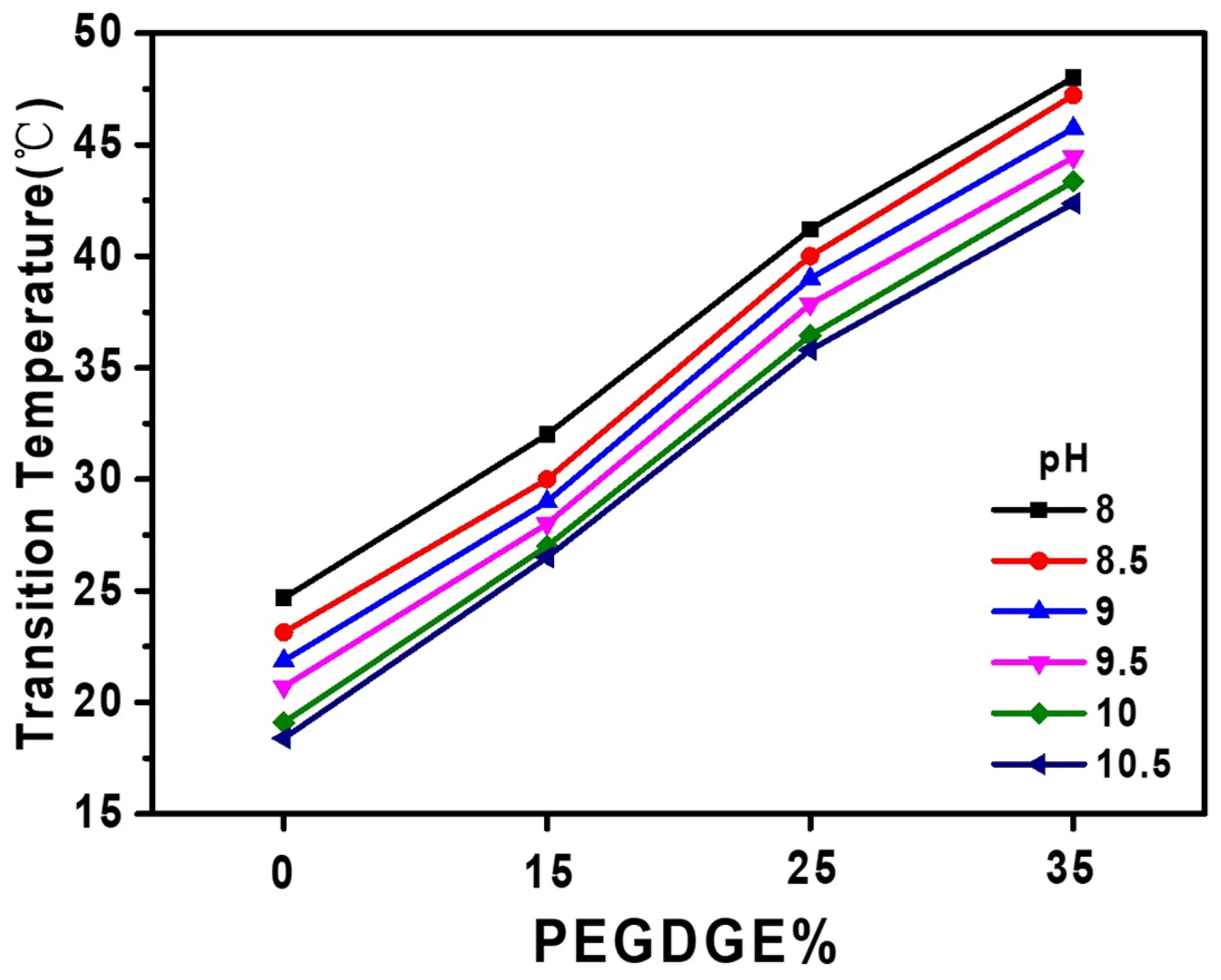
T_T_ and PEGDGE content dependence of PT-Gels with different pH.

Figure 6 and Figure 7 further confirm that T_T_ decreases with increasing pH and increases with increasing PEGDGE content.

### 2.4. Effect of NaCl on Phase Transition

**Figure 8:**
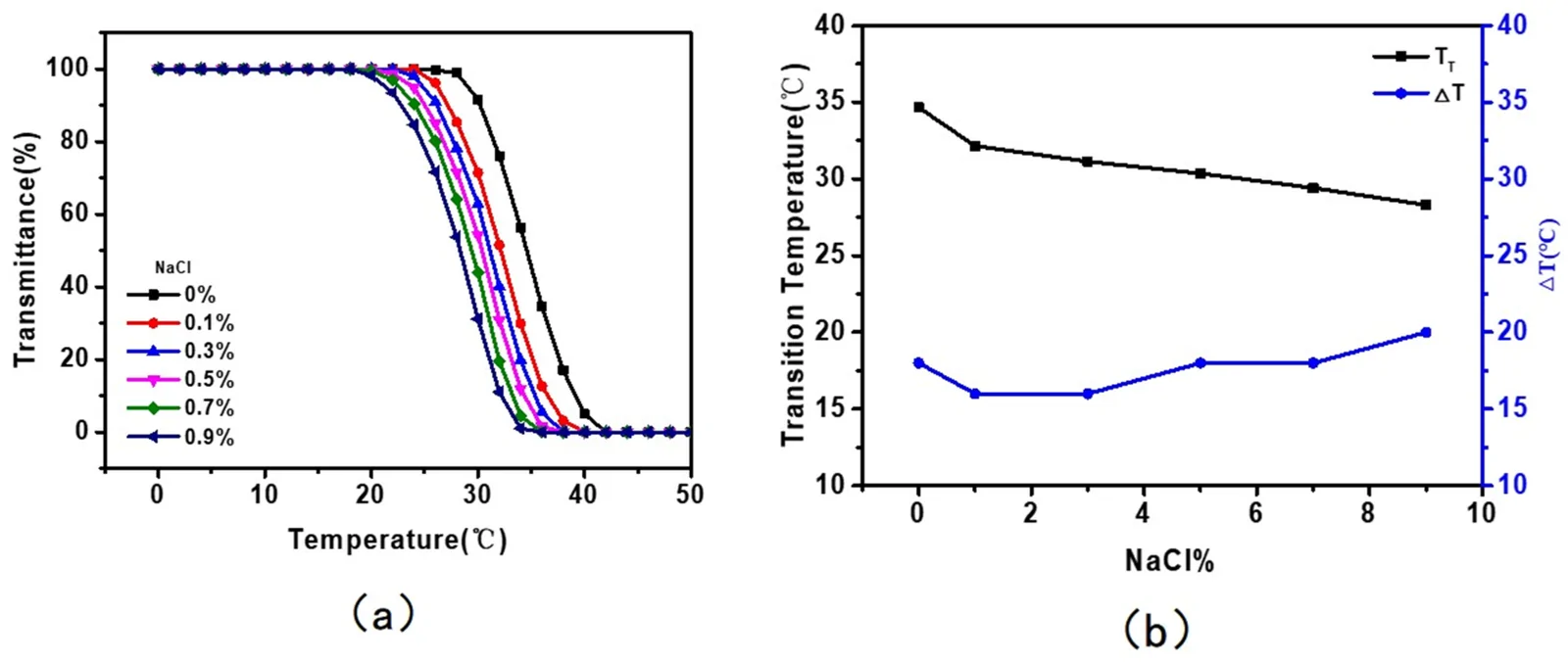
Effects of different NaCl doses on PT-Gels phase behavior: (**a**) dependence of optical transmittance on temperature for PT-Gels at different NaCl concentrations (0–0.9 wt%); (**b**) dependence of T_T_ on NaCl concentration

Figure 8 shows the influence of NaCl dosage. As NaCl concentration increases, T_T_ decreases. This is attributed to charge screening: Na^+^ and Cl^−^ ions shield the negative charges on the tertiary amines, reducing electrostatic repulsion and promoting hydrophobic collapse [50]. This salting-out effect is a common phenomenon in polyelectrolyte systems, where ions disrupt the hydration shell of the polymer chains, lowering the cloud point [51]. All NaCl-containing samples exhibit a ΔT within 20.5 °C, indicating a fast phase transition. Beyond the charge-screening effect, the observed NaCl-induced decrease in T_T_ can be further rationalized by considering specific ion (Hofmeister) effects and changes in polymer hydration. According to the Hofmeister series, Cl^−^ is classified as a chaotropic anion that can interact favorably with water molecules and directly with polymer chains, thereby disrupting the hydration shell around the hydrophobic PPGDGE segments [37,51]. This disruption reduces the energetic cost of dehydrating the polymer network, facilitating hydrophobic aggregation at lower temperatures and consequently lowering T_T_. Concurrently, the hydration of Na^+^ ions competes with polymer chains for water molecules, further diminishing the hydration layer around the hydrophilic PEGDGE segments and promoting chain collapse. These specific ion effects, combined with the charge-screening mechanism, provide a more comprehensive explanation for the salt sensitivity of PT-Gels and are consistent with recent reports on ion-specific effects in polyelectrolyte and amphiphilic polymer systems [38,40,51].

### 2.5. Effect of Urea on Phase Transition

**Figure 9:**
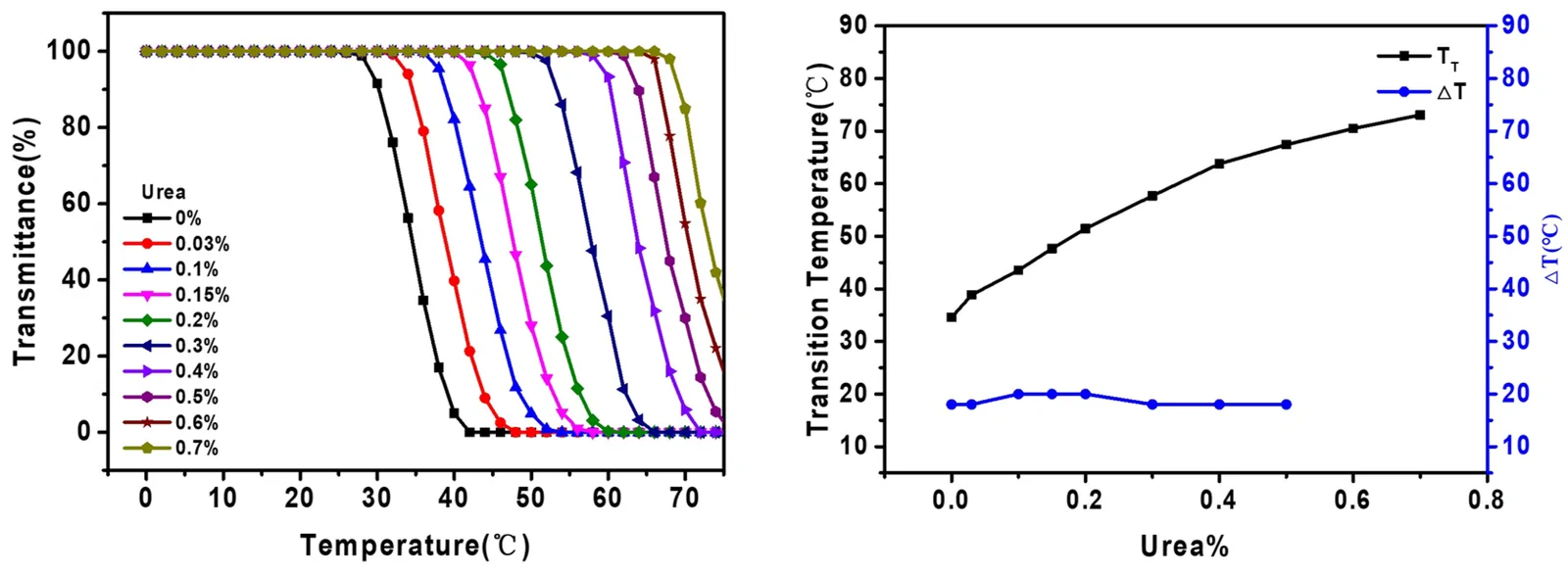
Effects of different urea doses on PT-Gel phase behavior.

Figure 9 displays the effect of urea. PT-Gels were synthesized via nucleophilic addition/ring-opening reactions between polyPEMPP prepolymer and T-403 crosslinker. The reaction of amino groups with epoxy groups generated abundant hydroxyl groups, enabling potential hydrogen-bond-like interactions. To verify hydrogen bonding, urea—a known hydrogen-bond disruptor [39,52]—was added to the initial reaction solution. As shown in Figure 9, increasing the urea content raised the T_T_ of PT-Gels. Without urea, T_T_ was ~34 °C; at 0.7 wt% urea, T_T_ exceeded 70 °C. A near-linear positive correlation between urea concentration and T_T_ was observed. These results confirm that hydrogen bonding promotes the phase transition shrinkage of PT-Gels, and urea inhibits this effect by raising T_T_ and impeding shrinkage [43,53,54]. This observation is consistent with recent reports on hydrogen bonding-driven hydrogels for motion monitoring applications [43], further supporting the critical role of hydrogen bonds in governing the responsive behavior of polymer networks. It is important to clarify that the observed urea-induced increase in T_T_ does not arise solely from direct hydrogen-bond disruption between polymer chains. Urea also exerts significant effects on the bulk water structure and polymer solvation. Specifically, urea molecules are known to break the tetrahedral hydrogen-bond network of water, thereby altering the solvation environment of both hydrophilic and hydrophobic segments [39,52]. This disruption of water structure reduces the hydrophobic hydration effect, making the aggregation of PPGDGE segments less thermodynamically favorable and thus requiring a higher temperature to induce phase transition. In addition, urea molecules can directly interact with the polymer chains via hydrogen bonding between urea’s amino groups and the hydroxyl groups generated from the epoxy–amine crosslinking reaction, as well as with ether oxygens along the polymer backbone. This direct urea–polymer interaction increases the solubility of the polymer network in an aqueous solution, further elevating T_T_ [43,53]. Therefore, the observed increase in T_T_ upon urea addition is a combined result of (1) the disruption of inter- and intra-chain hydrogen bonds within the polymer network; (2) the alteration of the bulk water structure that weakens hydrophobic hydration; and (3) enhanced polymer solvation through direct urea–polymer interactions. This multifaceted interpretation is consistent with recent studies on urea-induced denaturation and phase behavior in polymer systems [36,39,52].

### 2.6. Cyclic Phase Transition of PT-Gels

**Figure 10:**
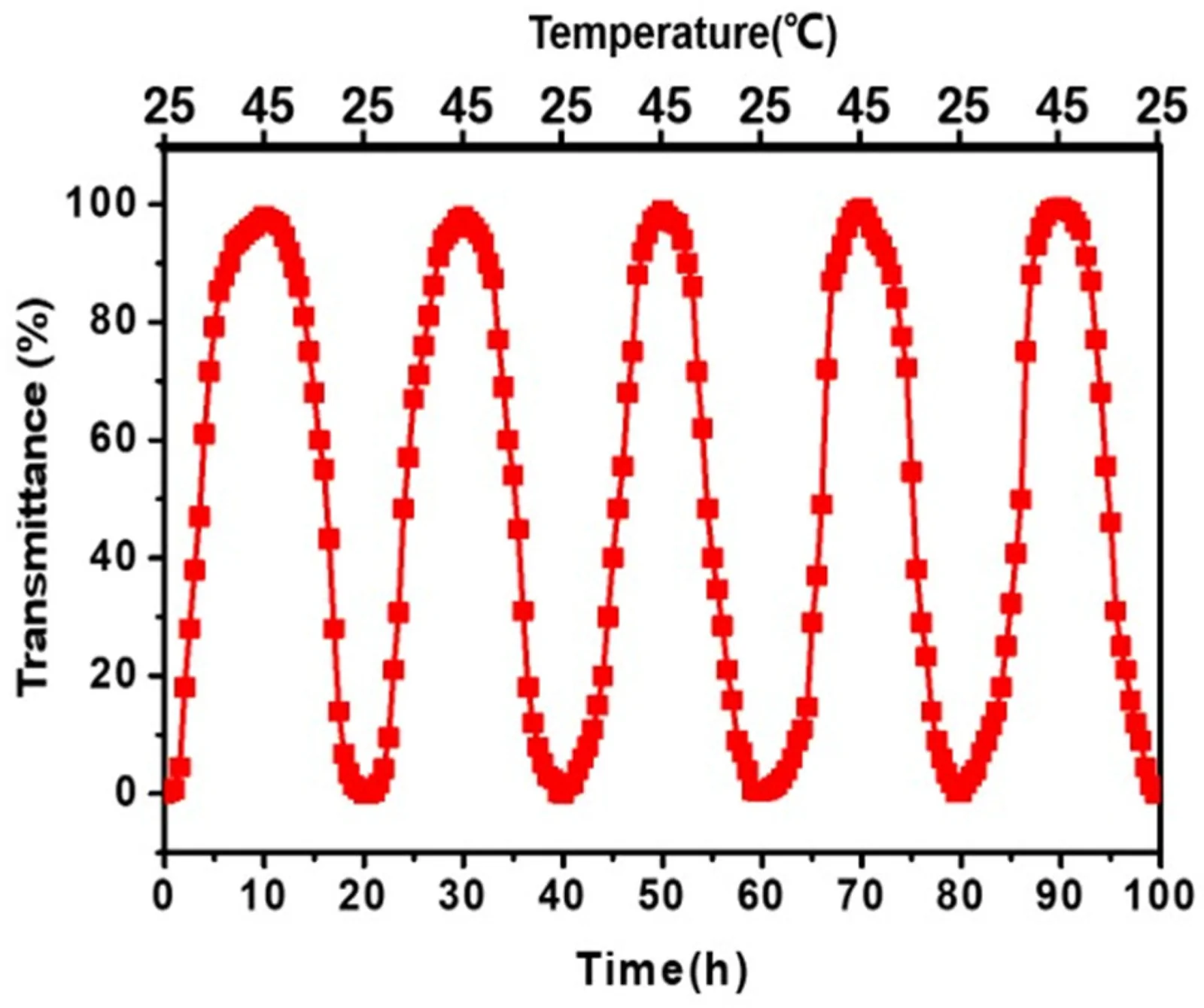
Oscillatory phase transition behavior of PT-Gels.

Figure 10 shows the oscillatory phase transition behavior of PT-Gels (sample with 15% PEGDGE). When cycled between 25 °C and 45 °C, the transmittance reversibly changes from 0% to 100% and back. After five cycles, the curves remain essentially unchanged, demonstrating excellent reversibility.

### 2.7. Mechanistic Understanding of the Phase Transition Behavior

Based on the systematic experimental results presented above, a comprehensive mechanistic picture emerges for the phase transition behavior of PT-Gels. The observed phase transition is governed by a delicate balance among three primary driving forces—hydrophobic interactions, electrostatic repulsion, and hydrogen bonding—which are in turn modulated by the network architecture of the crosslinked polymer system.

At the molecular level, the PPGDGE segments serve as the primary hydrophobic domains that drive chain collapse and aggregation at elevated temperatures. This thermoresponsive hydrophobic association is the initial trigger for the phase transition, consistent with classical LCST-type behavior observed in amphiphilic polymer systems [19,20,21,22,44]. However, unlike conventional PNIPAM-based hydrogels that rely predominantly on hydrophobic interactions alone, PT-Gels incorporate additional regulatory mechanisms that provide enhanced tunability.

The hydrophilic PEGDGE segments counteract hydrophobic collapse by maintaining chain hydration and increasing the overall hydrophilicity of the network. As demonstrated in Figure 1, increasing PEGDGE content systematically elevates T_T_ through a near-linear relationship, reflecting the competitive balance between hydration and dehydration forces. This composition-dependent tunability is a key advantage of our system, as it allows for the precise adjustment of T_T_ over a remarkably wide range (~12 °C to >75 °C) by simply varying the hydrophilic/hydrophobic balance [46,47].

To further strengthen the mechanistic basis for the pH-responsive behavior, the observed T_T_ changes can be directly correlated with the acid–base characteristics of the tertiary amine groups present in the PT-Gel network. The tertiary amine functionalities within the polyPEMPP backbone exhibit a pKa value in the range of approximately 8.0–10.5, which is characteristic of aliphatic tertiary amines. Consequently, at pH values below the pKa (e.g., pH 5.0–7.5), the amine groups are predominantly protonated, generating a high density of positive charges along the polymer chains. The resulting electrostatic repulsion not only inhibits hydrophobic aggregation—thereby significantly elevating T_T_—but also enhances chain hydration, further stabilizing the swollen state [45,48,49]. This protonation effect is most pronounced at pH 5.0, where the charge density reaches its maximum, corresponding to the highest observed T_T_ values (Figure 3). As the pH increases toward and above the pKa (e.g., pH 8.0–10.5), deprotonation progressively reduces the positive charge density, weakening electrostatic repulsion and allowing hydrophobic interactions to dominate, which consequently lowers T_T_. Importantly, the magnitude of the pH-induced T_T_ shift—approximately 4–6 °C per pH unit—is consistent with the degree of protonation change across this pH range, providing a quantitative link between the acid–base equilibrium and the macroscopic phase transition behavior. This pH-dependent electrostatic modulation is consistent with recent reports on charge-responsive hydrogels, where the pH sensitivity is directly governed by the pKa of the ionizable groups and the resulting electrostatic effects on network swelling and phase behavior [29,30,33]. The systematic relationship between pH and T_T_ observed in our study thus confirms that the tertiary amine functionalities serve as effective molecular switches, enabling the precise pH-controlled modulation of the phase transition through the protonation–deprotonation equilibrium.

Hydrogen bonding represents the third critical driving force. The crosslinking reaction between epoxy and amino groups generates abundant hydroxyl groups within the network, creating extensive hydrogen-bonding sites [50]. These hydrogen bonds contribute to network cohesion and promote chain association during the phase transition. The addition of urea, a well-known hydrogen-bond disruptor, effectively dismantles these interactions, raising T_T_ and impeding the shrinkage behavior [39,52]. This observation is consistent with recent studies demonstrating the crucial role of hydrogen bonding in governing the responsive behavior of polymer networks [43].

The network architecture, defined by the crosslinking density and the distribution of hydrophilic and hydrophobic segments, provides the structural framework within which these driving forces operate. The trifunctional T-403 crosslinker creates a three-dimensional chemically crosslinked network that restricts chain mobility and influences the cooperative nature of the phase transition. The long polyPEMPP chains between crosslinks allow for sufficient conformational freedom for hydrophobic segments to aggregate upon heating, while the crosslinks ensure macroscopic integrity and reversibility [44,45].

Importantly, these four factors do not act independently but operate synergistically. For instance, at low pH, the combined effects of electrostatic repulsion and enhanced chain hydration can overcome hydrophobic interactions even at elevated temperatures, resulting in the exceptionally high T_T_ values observed for high-PEGDGE-content samples (Figure 4). Conversely, at high NaCl concentrations, charge screening reduces electrostatic repulsion, allowing hydrophobic interactions to prevail at lower temperatures [50,51]. The excellent reversibility observed in Figure 10 confirms that the network architecture maintains its structural integrity through repeated cycling, with all three driving forces operating in a reversible manner. To contextualize the performance of PT-Gels within the broader field of multi-responsive hydrogels, we compared our system with representative previously reported hydrogels across several key parameters (Table 1). Collectively, this comparative analysis confirms that PT-Gels offer a compelling combination of synthetic simplicity, broad tunability, multi-stimuli responsiveness, excellent reversibility, and quantitative sensitivity, positioning them as a versatile platform for diverse applications.

In summary, the phase transition behavior of PT-Gels is governed by a synergistic interplay of hydrophobic interactions (driven by PPGDGE segments and temperature), electrostatic repulsion (modulated by pH), hydrogen bonding (influenced by urea), and network architecture (determined by crosslinking density and chain length). The ability to independently and simultaneously tune these factors within a single hydrogel system provides unprecedented control over the phase transition temperature and represents a significant advancement over existing multi-responsive materials [55,56]. This comprehensive mechanistic understanding not only explains our experimental observations but also provides a rational framework for designing next-generation smart hydrogels with precisely tailored responsive properties.

## 3. Conclusions

In this work, a novel multi-stimuli-responsive hydrogel (PT-Gel) was successfully synthesized via a one-pot crosslinking reaction between a linear polyPEMPP prepolymer and a trifunctional amine crosslinker (T-403). The T_T_ can be precisely tuned by varying the composition: increasing the hydrophilic PEGDGE content raises T_T_, while lowering pH also increases T_T_ due to electrostatic repulsion. Conversely, the addition of NaCl reduces T_T_ via charge screening, whereas urea increases T_T_ by disrupting hydrogen bonds.

These findings confirm that the phase transition is driven by a synergistic interplay of hydrophobic interactions, electrostatic effects, and hydrogen bonding [39,50]. Furthermore, the PT-Gels exhibit excellent reversible phase transition behavior, making them ideal candidates for controlled drug delivery, adaptive separation, and sensor systems. The rational design of hydrogels with tunable responses to multiple stimuli is a key trend in the development of smart materials, and this study provides a promising strategy for such applications [55,56].

## 4. Materials and Methods

### 4.1. Materials

The following chemicals were used as received without further purification: poly(ethylene glycol) diglycidyl ether (PEGDGE, chemical pure, Sigma-Aldrich, St. Louis, MO, USA), poly(propylene glycol) diglycidyl ether (PPGDGE, chemical pure, Sigma-Aldrich, St. Louis, MO, USA), ethanolamine (MEA, chemical pure, Tianjin Fuyu, Tianjin, China), trimethylolpropane tris(2-methyl-1-aziridinepropionate) (T-403, ≥99%, Hustman, TX, USA), anhydrous ethanol (chemical pure, Tianjin Fuyu, China), n-hexane (analytical grade, Tianjin Fuyu, China), hydrochloric acid (HCl, 32 wt%, Sigma-Aldrich, USA), sodium hydroxide (NaOH, ≥98%, Sigma-Aldrich, USA), sodium chloride (NaCl, ≥99.5%, Sigma-Aldrich, St. Louis, MO, USA), and urea (≥99.5%, Aladdin, Shanghai, China).

### 4.2. Instruments

Analytical balance (Me104, Millipore, Burlington, MA, USA), three-neck flask (GG-17, Sichuan Shubo Group, Chengdu, China), Buchner funnel (300 mm, Jiangsu San’aisi Science, Nanjing, China), rotary evaporator (RE-52, Gongyi Honghua Instrument Equipment, Gongyi, China), vacuum-drying oven (DZF-6020, Shenzhen Shanhe Company, Shenzhen, China), UV–Vis spectrophotometer (Shimadzu UV-2450, Kyoto, Japan), pH meter (Inlab @Micro, Chongqing Liuhui Technology, Chongqing, China).

### 4.3. Synthesis of PT-Gels

First, the prepolymer poly(poly(ethylene glycol) diglycidyl ether–monoethanolamine–poly(propylene glycol) diglycidyl ether) (polyPEMPPs) was synthesized via a one-pot condensation reaction between poly(ethylene glycol) diglycidyl ether (PEGDGE) and poly(propylene glycol) diglycidyl ether (PPGDGE) in the presence of ethanolamine (MEA) as a catalyst, following a previously reported procedure with minor modifications [57]. After synthesis, the crude polyPEMPPs were purified by precipitation in n-hexane and dried under vacuum at 50 °C for 12 h. Then, predetermined amounts of polyPEMPPs and crosslinker T-403 (see Table 2) were weighed according to the molar ratios specified for each sample series (the molecular weight of T-403 is approximately 420 g·mol^−1^). For the pH-dependent sample series (c-samples), HCl and NaOH solutions were pre-prepared at fixed molar concentrations and then finely adjusted to the target pH values using a pH meter before use. For other series, appropriate amounts of additives were added as follows: NaCl was added as a solid with concentrations ranging from 0 to 0.9 wt% relative to the total polymer mass (polyPEMPPs + T-403); urea was added as a solid with concentrations ranging from 0 to 0.7 wt% relative to the total polymer mass. Deionized water was added to adjust the solid content to 65 wt%, and the mixture was stirred for 3 h to ensure complete dissolution. Subsequently, the pre-adjusted HCl or NaOH solutions (for c-samples), or NaCl/urea (for d- and e-samples), were directly mixed with the polyPEMPP/T-403 mixture according to the formulations listed in Table 2. The mixture was stirred for another 30 min. For transmittance measurements, the solution was placed in a cuvette for in situ polymerization. The polymerization was carried out at 75 °C for 12 h to obtain PT-Gels.

### 4.4. Transmittance Measurement

PT-Gels polymerized in situ in a cuvette were placed in a UV–Vis spectrophotometer (Shimadzu UV-2450) equipped with a temperature controller. The wavelength was set to 600 nm. The temperature was increased from 0 °C to 75 °C with a step of 2 °C, and the transmittance was recorded after stabilizing for 15 min at each temperature. The cyclic phase transition of sample a-series with 15% PEGDGE was tested by alternately changing the temperature between 25 °C and 45 °C and recording the transmittance. All transmittance measurements were performed in triplicate using independently prepared samples. The reported T_T_ values represent the average of three independent measurements, and the data are presented as mean values. The temperature at which the transmittance reached 50% was defined as T_T_ [57], consistent with the convention used in analogous thermoresponsive hydrogel systems. The measurements were performed using a standard 10 mm path length quartz cuvette with a sample volume of approximately 3.0 mL. The wavelength of 600 nm was selected because it is a non-absorbing wavelength for the polymer system, ensuring that the observed transmittance changes reflect turbidity variations arising from phase separation rather than specific absorption by chromophores. This wavelength is widely used in turbidimetric studies of thermoresponsive polymer systems and provides a reliable measure of macroscopic phase transition behavior.

## Figures and Tables

**Table 1:** Comparison of PT-Gels with representative previously reported multi-responsive hydrogel systems.

Feature	PT-Gels (This Work)	PNIPAM-Based Hydrogels [19,20,21,22]	Schiff Base Hydrogels [34,35]	pH/Temperature Dual-Responsive Hydrogels [31,32]	Hydrogen Bonding-Driven Hydrogels [43,55,56]
Synthesis Strategy	One-pot crosslinking	Free-radical polymerization	Schiff base condensation	Multi-step or copolymerization	Multi-step assembly
Phase Transition Temp. (T_T_) Range	~12 °C to >75 °C	20–40 °C (LCST-type)	Not applicable (not thermoresponsive)	25–50 °C	Variable
Stimuli Responsiveness	Temperature, pH, NaCl, urea	Temperature (mainly)	pH, self-healing	Temperature, pH	Temperature, mechanical stress
Reversibility	Excellent (0% ↔ 100% over multiple cycles)	Good but limited cycling stability	Good (self-healing)	Moderate	Good
Sensitivity	Quantitative (linear correlations)	Qualitative	Qualitative	Quantitative (limited range)	Qualitative
Potential Applications	Drug delivery, sensors, separation, robotics	Drug delivery, tissue engineering	Wound healing, drug delivery	Drug delivery, biosensors	Wearable sensors, motion monitoring

**Table 2:** Raw material compositions.

Samples	PEGDGE%	T-403%	pH	NaCl%	Urea%
a-samples	0–35	11.1	7.7	0	0
b-samples	15	11.1–33.3	7.7	0	0
c-samples	15	11.1	5–10.5	0	0
d-samples	15	11.1	7.7	0–0.9	0
e-samples	15	11.1	7.7	0	0–0.7
f-samples	15	11.1	7.7	0	0

PEGDGE% = n(PEGDGE)/[n(PEGDGE) + n(PPGDGE)] × 100%. T-403% = n(T-403)/n(polyPEMPPs) × 100%. NaCl% = m(NaCl)/[m(polyPEMPPs) + m(T-403) + m(H_2_O)] × 100%. Urea% = m(Urea)/[m(polyPEMPPs) + m(T-403) + m(H_2_O)] × 100%.

## Data Availability

All data supporting the findings of this study are presented within the main manuscript and its figures. No other external datasets were generated or analyzed during this study.
